# Practicing what we preach for successful interprofessional education

**DOI:** 10.1111/tct.13433

**Published:** 2021-10-22

**Authors:** Juliëtte A. Beuken, Jascha de Nooijer, Renée E. Stalmeijer

**Affiliations:** ^1^ Department of Educational Development and Research, School of Health Professions Education Maastricht University Maastricht Netherlands; ^2^ Department of Health Promotion, School of Health Professions Education Maastricht University Maastricht Netherlands

While attending a workshop on moral deliberation at a national conference, I (Juliëtte, a health professions education researcher at a Medical School) sat next to a teacher from a Midwifery School. We started talking about how moral deliberation is an interprofessional affair. We reflected that midwifery and medical students could learn to discuss ethical dilemmas collaboratively. She and I set out to put our ideas into practice, reached out to our institutes, and discussed how we could design and organize interprofessional workshops on moral deliberation for our students.

Interprofessional education (IPE)—in which students from different professions learn with, from, and about each other—has been acknowledged as a key educational format to prepare health profession students for the interprofessional collaboration that healthcare requires. However, research has shown that one of the main premises underlying IPE, ‘just’ bringing students from different professions together, is not enough for them to develop competencies to collaborate well in future.^1^ Besides being a logistical challenge, IPE development, teaching and research require a shared effort by many stakeholders from different backgrounds, (i.e., healthcare professionals from different disciplines, education experts, teachers and students). Like many other developers, teachers and researchers in IPE, we acknowledge the necessity of this shared effort. However, we also see that our different backgrounds, concerns and interests also challenge IPE design. This dilemma was discussed in a special interest group meeting on IPE at our university and saw connections to a research methodology and a conceptual framework relevant in our work. Inspired by this, we propose two ways in which IPE can be strengthened through collaborative efforts by all IPE stakeholders: (1) by introducing a design‐based research approach^2^ to create a wider, theory‐ and practice‐informed evidence base for successful IPE and (2) by understanding IPE as working in a landscape of practice.^3^

IPE development, teaching and research require a shared effort by many stakeholders from different backgrounds.

First, theoretical design principles and practical experiences can inform IPE development, teaching and research. Generating a theory‐informed evidence base for IPE requires collaboration between those developing, teaching and researching IPE. *Design‐based research* (DBR), also known as *educational design research* (EDR), is a research methodology to collaboratively develop educational interventions and uses theoretical insights to solve practical problems in education. In DBR, stakeholders collaborate to analyse an educational problem, design education based on theory and practice and evaluate both the process and outcomes of education.^2^, ^4^ This approach results in both theoretical understanding *and* practical improvement of an educational design.^2^, ^4^ Through its combined purpose of building theory and informing practice, we believe DBR to be a suitable approach for the theory‐informed evidence base IPE needs. Moreover, by answering questions like ‘what works best under which circumstances’, and through its essentially collaborative nature, DBR could simultaneously boost a collaborative culture among developers, teachers and researchers.

As we designed and organized our workshop, power relations between midwifes and physicians were discussed. Teachers from both institutions voiced that midwifes sometimes feel unheard by physicians, while physicians may feel the responsibility to take charge. This discussion reminded me of literature we discussed in our special interest group about boundaries between professions. This literature described how different professionals sometimes have trouble empathizing with other professionals' views—a central concept in moral deliberation—because they are unaware of each other's unique contributions to healthcare. The literature suggested that voicing these unique contributions of different stakeholders might help to overcome such barriers. We combined theory and practical experience to create awareness of different professional contributions by asking additional questions for some components of our workshop on moral deliberation (e.g. ‘how do you expect others to act in a situation and what makes them act like that?’).

I Generating a theory‐informed evidence base for IPE requires collaboration between those developing, teaching and researching IPE.

Second, IPE design often requires stakeholders from different *Communities of Practice* (CoP) to learn how to navigate the IPE *Landscape of Practice* (LoP),^3^ a metaphor for multiple CoP and the boundaries between them. When teachers, designers and researchers collaborate, we encounter different views on IPE (e.g., what we consider appropriate content, methods, and goals). To make these differences work for instead of against IPE, stakeholders need to look further and cross the boundaries of their own CoP into the LoP.^3^, ^5^ This requires *knowledgeability*: the ability to recognise the value of members from different CoPs and one's own to IPE.^3^, ^5^ In collaborations between knowledgeable members of the LoP, stakeholders can learn to understand that seemingly competing interests of those developing, teaching and researching IPE will eventually contribute to the same goal: competent future health professionals.

In our discussions about the workshop we were designing, I realized we were not always on the same page. Every professional at the table, teachers, designers, and researchers, had different ideas about what was important and what the workshop should look like. For example, I wanted a structured evaluation that focused on the design of the workshop, something I had just learnt to do in my own educational research. Others, teachers with years of experience, wanted a more informal evaluation that focused on the students' reflections. Reasoning from our own professions and informed by our own experiences, we had different opinions about goals of and appropriate methods for evaluation. We discussed our different perspectives on evaluation, how different approaches would benefit our shared goal (students' interprofessional learning experience), and made concessions in the design of the workshop accordingly.

Seemingly competing interests of those developing, teaching and researching IPE will eventually contribute to the same goal.

In the end, successful IPE requires those developing, teaching and researching to value and use insights from each other's respective professions and disciplines, and develop the same interprofessional collaborative competences we require of our students. Using DBR can be an approach to not only bring those involved in IPE closer together but to also strengthen the field of IPE by more effectively engaging in the LoP we all work in. Ultimately, we need to practice what we preach.

## CONFLICT OF INTEREST

The authors have no conflict of interest to disclose.

## ETHICAL APPROVAL

No ethical approval was required for this work.

## References

[1] Paradis E , Whitehead CR . Beyond the lamppost: a proposal for a fourth wave of education for collaboration. Acad Med. 2018;93(10):1457–1463. 29620672 10.1097/ACM.0000000000002233 PMC6159689

[2] Dolmans DH , Tigelaar D . Building bridges between theory and practice in medical education using a design‐based research approach: AMEE Guide No. 60. Med Teach. 2012;34(1):1–10. 22250671 10.3109/0142159X.2011.595437

[3] Wenger‐Trayner E , Wenger‐Trayner B . Learning in a landscape of practice: a framework. In: Learning in landscapes of practice. London: Routledge; 2014. p. 13–29.

[4] McKenney S , Reeves TC . Educational design research: portraying, conducting, and enhancing productive scholarship. Med Educ. 2021;55(1):82–92. 32564385 10.1111/medu.14280 PMC7754301

[5] Hodson N . Landscapes of practice in medical education. Med Educ. 2020;54(6):504–9. 31953968 10.1111/medu.14061

